# An *In Vitro* Fermentation Study on the Effects of Gluten Friendly^TM^ Bread on Microbiota and Short Chain Fatty Acids of Fecal Samples from Healthy and Celiac Subjects

**DOI:** 10.3389/fmicb.2017.01722

**Published:** 2017-09-07

**Authors:** Adele Costabile, Triana Bergillos-Meca, Loretta Landriscina, Antonio Bevilacqua, Isidro Gonzalez-Salvador, Maria R. Corbo, Leonardo Petruzzi, Milena Sinigaglia, Carmela Lamacchia

**Affiliations:** ^1^Health Science Research Centre, Department of Life Sciences, Whitelands College, University of Roehampton London, United Kingdom; ^2^Department of the Science of Agriculture, Food and Environment, University of Foggia Foggia, Italy

**Keywords:** Gluten Friendly bread, fecal microbiota, celiac, healthy, gut model

## Abstract

Recently, an innovative gluten detoxification method called Gluten Friendly^TM^ (GF) has been developed. It induces structural modifications, which abolish the antigenic capacity of gluten and reduce the *in vitro* immunogenicity of the most common epitopes involved in celiac disease, without compromising the nutritional and technological properties. This study investigated the *in vitro* effects of GF bread (GFB) on the fecal microbiota from healthy and celiac individuals by a three-stage continuous fermentative system, which simulates the colon (vessel 1, proximal colon; vessel 2, transverse colon; and vessel 3, distal colon), as well as on the production of short chain fatty acids (SCFA, acetate, propionate, butyrate). The system was fed with GFB and the changes in microbiota through fluorescence *in situ* hybridization and in SCFA content were assessed. GFB exerted beneficial modulations such as bifidogenic effects in each compartment of the model both with healthy- and celiac-derived samples, as well as growth in *Clostridium* clusters XIVa+b in celiac-derived samples. Furthermore, increased levels of acetic acid were found in vessel 1 inoculated with the fecal microbiota of healthy individuals, as well as acetic and propionic in vessel 1 and 2 with celiac-derived samples. In addition, the use of multivariate approaches showed that the supplementation of GFB could result in a different modulation of the fecal microbiota and SCFA, as a function of initial equilibrium.

## Introduction

The composition and the metabolism of human microbiota play crucial roles in human health. Microbial colonization of the gastrointestinal tract varies widely, with the large intestine having not only the highest density of microbes in terms of bacterial cells *per* gram but also the most metabolically active organ (Turnbaugh et al., 2006). Genetics, mode of birth, infant feeding patterns, antibiotic usage, sanitary living conditions, and long-term dietary habits contribute to shaping the composition of the gut microbiome. Diet clearly has been considered as a major driver for changes in the compositional and functional relationship between microbiota and the host (Muegge et al., 2011). The fermentative but also (anaerobic) respiratory bacterial metabolism of dietary components produces an extraordinary chemical diversity in the large intestine with protective (e.g., short chain fatty acids [SCFAs]) or detrimental (e.g., hydrogen sulfite, phenol, *p*-cresol, or bile acids) effects on the disease development (Flint, 2012). There have been numerous attempts to identify a “core” microbiota, usually defined as bacterial taxa that are shared between 95% of individuals tested. Identification of a core microbiome is important for defining a “normal” healthy state from which major variations may indicate a dysbiotic system that can result from or contribute to disease development.

Recent reports have shown how the intestinal microbiota in celiac patients might be altered and influenced underlying mucosal immune response. Celiac disease (CD) is a chronic immune-mediated enteropathy triggered by the ingestion of gluten, the water-insoluble protein fraction in wheat, rye, and barley, in patients who are HLA-DQ2 or HLA-DQ8 positive. Approximately 30% of the general population carry the HLA-DQ2/8 CD susceptibility genes; however, only 2–5% of these individuals will go on to develop CD, suggesting that additional environmental factors contribute to disease development. Generally, gluten-free diet alleviates many of the symptoms, but somewhat surprisingly, it does not completely restore healthy microbiota profiles (Wacklin et al., 2014).

Recently, a new and innovative gluten detoxification method has been developed (PCT/IB2013/000797) (Lamacchia et al., 2013, 2015a). It is usually referred to as Gluten Friendly^TM^ (GF) and lies in the application of microwave energy for few seconds to hydrated wheat kernels. This technology induces structural modifications to endosperm components which abolish the antigenic capacity of gluten and dramatically reduce *in vitro* the immunogenicity of the most common epitopes involved in CD (Lamacchia et al., 2015b, 2016), without compromising both the nutritional and technological properties necessary to process flour into bread, pasta, and other baked goods. Recently, Landriscina et al. (2017) confirmed the effect of this new technology on the immunogenic epitopes. They combined microscopy with immunodetection with specific antibodies for gliadins, γ-gliadins, LMW subunits, and antigenic epitopes to gain a better understanding of the technology at a molecular level. Cross-reactivity toward the antibodies recognizing almost the entire range of gluten proteins as well as the antigenic epitopes through the sequences QQSF, QQSY, PEQPFPQGC, and QQPFP was significantly reduced.

This study aimed to investigate the impact of the administration of GF bread (GFB) on the fecal microbiota of healthy and celiac volunteers using a three-stage continuous culture colonic model system. This is a useful tool to monitor the ecology and metabolic activities of the microbiota in the proximal, transverse, and distal colon, in relation to different environmental conditions, dietary intervention, and the administration of drugs and antimicrobials. The influence of GFB on the fecal microflora and SCFA production [acetate, propionate (PRO), butyrate] was determined.

## Materials and Methods

### Substrate

Grain of treated wheat (according to the patented method PCT n. PCT/IB2013/000797) was milled commercially and kindly supplied by Casillo Group S.p.A^1^. The treatment was performed as follows: 100 g of cleaned wheat grains was dampened until reaching 15–18% humidity, which was measured by a halogen thermal balance (Mettler Toledo, HB43-S, Switzerland), and subjected to rapid heating via microwaves (Delonghi, Italy; approximately 1 min between 1000 and 750 watts), followed by slow evaporation of the water. The rapid heating and subsequent slow evaporation of the water was repeated until reaching a final temperature of 80–90°C, which was measured by a thermal camera (Fluke, i20 Model, Italy), and a moisture degree of 13–13.5% in the wheat grains.

After microwave treatment, the wheat kernels were cooled and dried at room temperature (24°C) for 12–24 h and then ground using an automatic laboratory mill MCKA (Bühler AG, Uzwil, Switzerland; diameter of grid 118–180 μm) (Bevilacqua et al., 2016).

Gluten Friendly bread was prepared according to the bread-making process (100 g wheat flour, 66 mL water, 1.33 g yeast, 1 g salt) to the Chorleywood Bread Process (Bevilacqua et al., 2016).

### Simulated *In Vitro* Human Digestion

Prior to being added to the *in vitro* colonic system, GFB was digested *in vitro*, under appropriate conditions according to the procedures described by Maccaferri et al. (2012) to mimic mouth, stomach, and intestine’s condition.

### Fecal Samples Collection and Inocula Preparation

Fecal samples were obtained from two healthy and two celiac donors (male and female aged 30–50 years old) who were free of any metabolic and gastrointestinal diseases, were not taking probiotic or prebiotic supplements, and had not taken antibiotics 6 months before fecal sample donation. All donors have provided written informed consent and filled in a standard questionnaire to collect information regarding health status, drug use, clinical anamnesis, and lifestyle factors. The University of Reading Research Ethics Committee (UREC 15/20) approved this study in accordance with the Declaration of Helsinki. Sample size was in accordance with previous studies (Bevilacqua et al., 2016). Fecal samples were placed in an anaerobic jar (AnaeroJar^TM^ 2.5 L, Oxoid Ltd.) including a Gas-Generating Kit (AnaeroGen^TM^, Oxoid Ltd.) in order to reproduce anaerobic conditions inside the chamber. An aliquot of 20 g of samples was diluted in 100 mL anaerobic PBS (0.1 mol/L phosphate buffer solution, pH 7.4, w/w) and homogenized (Stomacher 400; Seward, West Sussex, UK) for 2 min at 240 paddle beats per minute. Samples were added to anaerobic fermenters within 15 min of voiding.

### Three Stage-Continuous Culture Gut Model System

**FIGURE 1:**
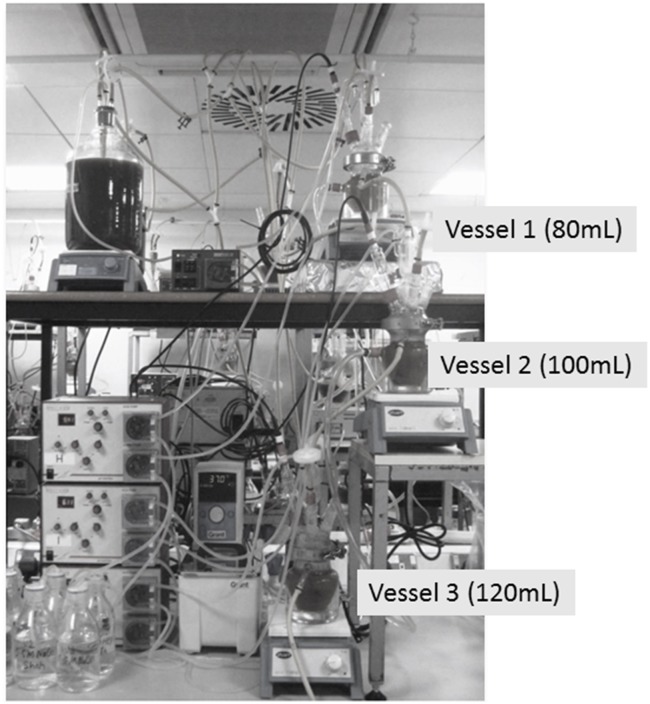
Schematic chart of gut model system used in this research.

Physicochemical conditions in the colon were replicated in a three-stage continuous system, comprised of a cascade of three glass fermenters of increasing working volume connected in series. A small-scale version of the validated system by Macfarlane et al. (1998) has been used in this study, as suggested by Grimaldi et al. (2017). The gut model is often used to resemble the complexity and diversity of the intestinal microbiota and simulates the proximal [vessel 1 (V1), 80 mL, pH 5.5], transverse [vessel 2 (V2), 100 mL, pH 6.2], and distal colon [vessel 3 (V3), 120 mL, pH 6.8] (**Figure 1**); the vessels were filled with fecal homogenate from healthy and celiac volunteers (20%) and a complex colonic growth medium (80%). The growth medium contained the following ingredients: starch, 5 g/L; mucin, 4 g/L; casein, 3 g/L; peptone water, 5 g/L; tryptone water, 5 g/L; bile salts, 0.4 g/L; yeast exact, 4.5 g/L; FeSO_4_, 0.005 g/L; NaCl, 4.5 g/L; KCl, 4.5 g/L; KH_2_PO_4_, 0.5 g/L; MgSO_4_ × 7H_2_O, 1.25 g/L; CaCl_2_ × 6H_2_O, 0.15 g/L; NaHCO_3_, 1.5 g/L; Tween 80, 1 mL; hemin, 0.05 g/L; and cysteine HCl, 0.8 g/L. Following inoculation, the colonic model was run as a batch culture for 24 h to stabilize the bacterial populations prior to the initiation of medium flow. After 24 h (*T*_0_), the medium flow was initiated and the system was ran for eight full volume turnovers to allow for steady state to be achieved (SS1) assessed through stabilization of the SCFA profiles (±5%); the duration of each turnover was 48 h, thus this first step was done for 384 h. The flow rate of the system was 6.25 mL/h. The system was run for further eight volume turnovers upon which steady-state 2 (SS2) was achieved; daily 3.75 mL of digested GFB was added. Each steady state was confirmed by stabilization of SCFAs profiles over 3 consecutive days. SS2 was achieved after 384 h; the whole experiments took place for 792 h. Each vessel was magnetically stirred and continually sparged with oxygen free nitrogen gas. Temperature (37°C) was maintained by a water-cooling system and culture pH was controlled automatically through the addition of 1 N NaOH or HCl. Aliquots of 4.5 mL were removed at SS1 and SS2 from each vessel (V1, V2, and V3).

### Short Chain Fatty Acids (SCFAs) Analysis by HPLC

The production of acetic, propionic, and butyric acids in the fermentations was determined by HPLC (Merck, NJ, United States) equipped with RI detection. The column used was an ion-exclusion REZEX-ROA organic acid column (Phenomenex Inc., United Kingdom) and temperature maintained at 84°C. Sulfuric acid in HPLC-grade H_2_O (0.0025 mmol/L) was used as the eluent, and the flow rate was maintained at 0.5 mL/min. Aliquots of 1 mL collected from each vessel in microcentrifuge tubes were centrifuged at 1136 × *g* for 10 min to remove all particulate matter. The supernatants were then filtered using 0.22 μm low protein binding Durapore polyvinylidene fluoride (PVDF) membranes (Millex; EMD Millipore, Billerica, MA, United States). Twenty microliters of each sample was injected with a run of 45 min. Peaks were integrated using the Atlas Lab Managing Software (Thermo Lab Systems, Mainz, Germany). Quantification of the samples was obtained through calibration curves of acetic, propionic, and butyric acids in concentrations ranging between 12.5 and 100 mM.

### *In Vitro* Enumeration of Bacterial Populations by Fluorescence *In Situ* Hybridization (FISH) Analysis

Fluorescence *in situ* hybridization analysis was performed as described by Costabile et al. (2014). Briefly, aliquots (375 μL) of gut model samples were fixed in three volumes of ice-cold 4% (w/v) paraformaldehyde for 4 h at 4°C. They were then centrifuged at 13,000 × *g* for 5 min and washed twice in 1 mL of sterile PBS. The cells were again pelleted by centrifugation and re-suspended in 150 μL of sterile PBS, to which 150 μL of ethanol was added. Samples were then vortexed and stored at -20°C until used in hybridizations. For the hybridizations, all probes were commercially synthesized and 5′-labeled with the fluorescent dye (Sigma-Aldrich, St. Louis, MO, United States). The probes used in this study are detailed in **Table 1**.

**Table 1:** FISH oligonucleotide probes used in this study.

Probe	Target bacterial group/species	Target sequence (5′–3′)	Hybridization/washing *T* (°C)	Reference
Bifl64	*Bifidobacterium* spp. (BIF)	CATCCGGCATTACCACCC	50–50	Langendijk et al., 1995
Erec482	*Clostridium* clusters XIVa+b (ERE)	GCTTCTTAGTCARGTACCG	50–50	Franks et al., 1998
Labl58	*Lactobacillus/Enterococcus* spp. (LAB)	GGTATTAGCAYCTGTTTCCA	50–50	Harmsen et al., 1999
Chisl50	*Clostridium histolyticum* group clusters I, II (CHI)	TTATGCGGTATTAATCTYCCTTT	50–50	Franks et al., 1998
Bac303	*Bacteroides–Prevotella* group (BAC)	CCAATGTGGGGGACCTT	46–48	Manz et al., 1996
Eub338 I^∗^	Most bacteria (EUB)	GCTGCCTCCCGTAGGAGT	46–48	Daims et al., 1999
Eub338 II^∗^	Most bacteria (EUB)	GCAGCCACCCGTAGGTGT	46–48	Daims et al., 1999
Eub338 III^∗^	Most bacteria (EUB)	GCTGCCACCCGTAGGTGT	46–48	Daims et al., 1999


*^∗^These probes were used in equimolar concentrations (50 ng/mL). Formamide (35%) was included in the hybridization buffer.*

### Statistical Analysis

The analyses were performed on two independent batches (two healthy donors and two celiac subjects); for each batch, the experiments were done in triplicate. Bacterial counts and SCFAs were statistically analyzed using paired *t*-tests to assess the effect of the same treatment at different time points. Significant differences were defined at *p* < 0.05. All analyses were performed using GraphPad Prism 6.0.

Furthermore, FISH and SCFAs data were standardized and reported as increase/decrease of SS2 (steady state at the end of the assay) relative to SS1 (steady state before the addition of GFB); each donor was treated as a separate “sample.”

First, a principal component analysis was run by using all SCFA and microbial groups as leading variables. The analysis was done following the traditional approach of PCA to qualitatively study the effect of the supplementation of GFB on each donor in each vessel.

Thereafter, the PCA was modified and run through the approach of the Multivariate Statistical Process Control/NIPALS algorithm (non-linear iterative partial least square); the number of interactions was set to 50. For this second analysis, the leading variables were either standardized SCFA or standardized microbial counts. The main outputs of this second approach are the standardized plots, showing the cases (i.e., each donor per each vessel), the line of the leading variables (the microbial groups for the first analysis and the SCFAs for the second analysis) and for each variable the minimum and maximum values. The benefit of this approach is that it allows the reader to point out both a qualitatively and a quantitatively grouping of cases. PCA and modified PCA were done through the software Statistica for Windows, ver. 12.0 (Statsoft, Tulsa, OK, United States).

## Results

### Bacterial Enumeration and SCFA

**FIGURE 2:**
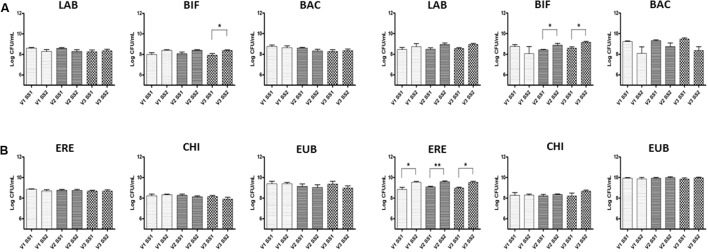
Bacterial groups in the culture broth recovered from the three different vessels (V1, V2, and V3) of the colonic model system before (SS1) and after (SS2) GFB supplementation. Inoculation with fecal slurries from healthy **(A)** and celiac donors **(B)**. Results are reported as means of the data of two models ± SEM (*n* = 2). SS1, steady state 1; SS2, steady state 2; GFB, Gluten Friendly bread; LAB, *Lactobacillus*–*Enterococcus* (Lab158); BIF, bifidobacteria (Bif164); BAC, *Bacteroides*–*Prevotella* (Bac303); ERE, *Clostridium* clusters XIVa+b (Erec482); CHI, *Clostridium* group clusters I, II (Chis 150); and EUB, bacteria (Eub 338 I, 338 II, 338 III).

Changes in the bacterial composition are in **Figure 2**; these results focused on the global effect of GFB, without considering a possible variability linked to donors. A significant increase in numbers of bacteria within the *Bifidobacterium* genus was found; fecal microbiota of celiac donors showed a significant increase in this population from 8.42 to 8.90 log CFU/mL (*p* < 0.05) and from 8.60 to 9.20 log CFU/mL (*p* < 0.05) in V2 and V3, respectively, transverse and distal colon. In the fecal samples of healthy volunteers, there was a significant increase in numbers of bifidobacteria (BIF) in V3, from 7.90 to 8.40 log CFU/mL (*p* < 0.05). Significant increases were also found in numbers of bacteria in all vessels for celiac volunteers in *Clostridium* cluster population, from 8.85 to 9.50 (V1), from 9.1 to 9.60 (V2), and from 9.00 to 9.50 log CFU/mL (V3) (*p* < 0.05) (ERE).

**FIGURE 3:**
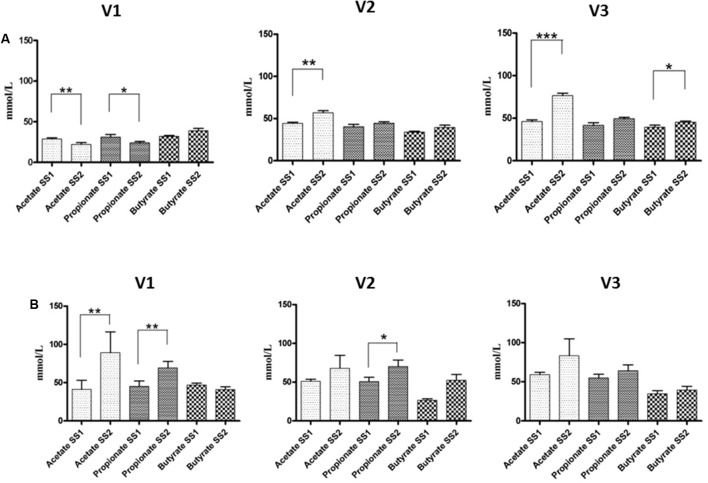
SCFA concentrations in the culture broths recovered from the three different vessels (V1, V2, and V3) of the colonic model system before (SS1) and after (SS2) GFB supplementation. Inoculation with fecal slurries from healthy **(A)** and celiac donors **(B)**. Results are reported as means of the data of two models ± SEM (*n* = 2). SS1, steady state 1; SS2, steady state 2; GFB, Gluten Friendly bread.

The results of SCFAs are in **Figure 3**. The fermentation of GFB by the fecal microbiota of healthy donors showed significant differences of acetic acid in all vessels, from 28.80 to 22.10 (V1) (*p* < 0.01), from 44.40 to 56.94 (V2) (*p* < 0.01), and from 46.00 to 76.50 mM (V3) (*p* < 0.001), respectively; butyrate also increased. Regarding the fecal microbiota of celiac volunteers, significant increases of propionic production were found in V1 and V2, from 45.10 to 69.20 (*p* < 0.01) and from 50.80 to 70.20 mM (*p* < 0.05), respectively; also, acetic acid showed a significant increase in V1, from 41.20 to 89.00 mM (*p* < 0.01).

### Multivariate Approach

**FIGURE 4:**
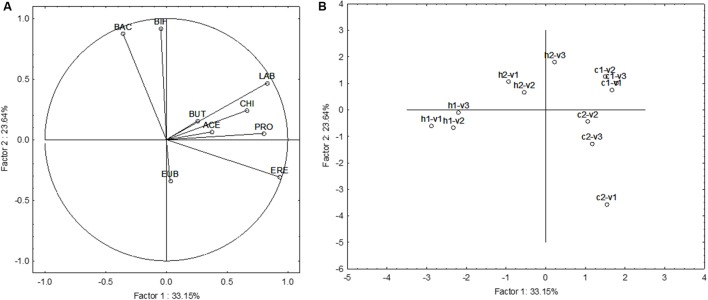
Principal Component Analysis on the increase of FISH counts and SCFA after the supplementation of GFB; the gut models were inoculated with the fecal slurries from healthy (letter H) and celiac donors (letter C). V1, V2, and V3, vessels of the colonic model and 1 and 2, donors 1 or donors 2; LAB, *Lactobacillus*–*Enterococcus* (Lab158); BIF, bifidobacteria (Bif164); BAC, *Bacteroides*–*Prevotella* (Bac303); ERE, *Clostridium* clusters XIVa+b (Erec482); CHI, *Clostridium* group clusters I, II (Chis 150); EUB, bacteria (Eub 338 I, 338 II, 338 III); BUT, butyrate; ACE, acetate; and PRO, propionate. **(A)** The projection of the variables, whereas **(B)** reports case projection.

The last approach was a focus on the multivariate differences induced by the supplementation of GFB. The results were reported as increase/decrease in FISH, and SCFAs (difference SS2-SS1). **Figure 4** shows the Principal Component Analysis on FISH and SCFA variations; all acids and microbial counts were used as leading variables. A difference from the previous approach (**Figures 2**, **3**) was the use of each donor as a separate sample to highlight the differences due to the different fecal microbiota (effect of “donor”). **Figure 4A** shows the projection of the leading variables. Bifidobacteria (BIF) and *Bacteroides*/*Prevotella* (BAC) were mostly related to the factor 2 (the correlation coefficients were 0.918 and 0.874, respectively), whereas *Lactobacillus*/*Enterococcus* (LAB), *Clostridium* (EREC), and propionate (PRO) were related to the factor 1 (correlation coefficients ranging from 0.804 to 0.934). This first PCA suggests an effect of GFB on the whole ecosystem (fecal microbiota and produced acids); however, this effect could be also affected by the initial situation of the system, in this analysis expressed as “the donor.” GFB exerted a strong effect on the fecal microbiota of the celiac donor 2, as suggested by the shift from c2–v1 (acids and fecal microbiota of the celiac donor 2 in the first vessel) to c2–v2 and c2–v3. The increase of BIF played a major role for this increase. On the other hand, the same effect was not found in the vessels inoculated with the fecal microbiota of the celiac donor 1. A modification of the system was also found in the vessels inoculated with the fecal microbiota of healthy donor 1, as a slight shift toward right was found (from v1 to v2–v3).

This first approach suggests that GFB could induce a shift and a modification of the ecosystem; however, due to the high number of the leading variables (nine), the accounted variability explained by the statistic is quite low (ca. 57%). Therefore, a second approach was used by using either the data of FISH and on SCFA; in addition, PCA was modified with NIPALS algorithm to achieve quantitative data for each vessel and see what is the effect of the supplementation on the evolution from V1 to V2 and V3 and simulate what could happen from a region to another of the colon.

**FIGURE 5:**
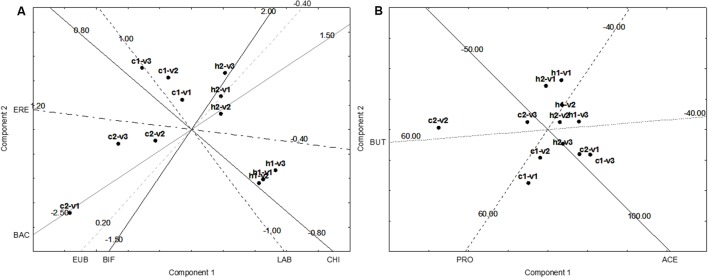
Principal Component Analysis on the increase of FISH counts and SCFA after the supplementation of GFB; the gut models were inoculated with the fecal slurries from healthy (letter H) and celiac donors (letter C). V1, V2, and V3, vessels of the colonic model; 1 and 2, donors 1 or donors 2. The numbers on the projection of the leading variables represent the quantitative data (minimum, central point, or maximum). **(A)** Effect on FISH counts. LAB, *Lactobacillus*–*Enterococcus* (Lab158); BIF, bifidobacteria (Bif164); BAC, *Bacteroides*–*Prevotella* (Bac303); ERE, *Clostridium* clusters XIVa+b (Erec482); CHI, *Clostridium* group clusters I, II (Chis 150); and EUB, bacteria (Eub 338 I, 338 II, 338 III). **(B)** Effect on SCFA. BUT, butyrate; ACE, acetate; PRO, propionate. **(A)** The projection of the variables, whereas **(B)** reports case projection.

**Figure 5A** shows the results for the FISH. In the fecal microbiota of the healthy donor 1, the effect of the supplementation could be seen in the vessel 1, without differences from the vessels 2 and 3. On the other hand, the fecal microbiota of the healthy donor 2 experienced some differences, as from V1 to V2–V3 there was an effect on BIF, with a slight increase (ca. 0.4 log CFU/mL).

The most important differences were found for the fecal microbiota of the celiac donors: in the donor 2, there was a shift from vessel 1 to vessels 2 and 3, probably due to BIF. In the vessel 1, there was a decrease of BIF (ca. 1 log CFU/mL), while in the vessels 2 and 3 BIF were restored with a mean decrease of 0.0–0.2 log CFU/mL.

The effect on the fecal microbiota of the celiac donor 1 was different. A slight effect on BIF was found in the vessel 1 and maintained in the vessels 2 and 3 (0.5–0.8 log CFU/mL), but a shift was found due to the different effect on *Lactobacillus*/*Enterococcus* and *Clostridium* cluster XIVa+b, as in the vessel 1 the increase was not significant (0.2 log CFU/mL), but in the vessel 3 the mean increase was 0.6–0.8 log CFU/mL. The difference in lactobacilli population was not significant when the mean between the two donors was done; on the other hand, a preliminary *t*-test revealed that on the donor 1 it was significant (*p* < 0.01).

**Figure 5B** shows the NIPALS approach for the SCFA; the main difference was in c2–v2 (vessel 2 inoculated with the fecal microbiota of the celiac donor 2). In this vessel, there was a strong increase of butyrate (ca. 45–50 mmol/L).

## Discussion

The human colon contains a wide range of bacterial communities, distributed in hundreds of distinct species, and the balance among them plays an important role in health and disease (Holzapfel et al., 1998; Rigottier-Gois et al., 2003). Although a consensus for what constitutes a core gut microbiome has been elusive, one report suggested that an international cohort of 39 individuals could be assigned to one of three distinct clusters or “enterotypes” based on metagenomic sequences. Arumugam et al. (2011) have found that each cluster was dominated by a particular bacterial genus (*Bacteroides, Prevotella*, and *Ruminococcus*) with positive or negative associations with a number of other genera in the community. Many authors have found differences in the composition of the intestinal microbiota between CD patients and healthy volunteers (Collado et al., 2007; Sanz et al., 2007; Di Cagno et al., 2009; De Palma et al., 2010; Nistal et al., 2012; Sellitto et al., 2012). In fact, CD patients frequently present altered intestinal bacterial groups such as *Bifidobacterium*, *Lactobacillus*, *Bacteroides*, *Prevotella*, *Staphylococcus*, and *Escherichia* (Collado et al., 2007; Sanz et al., 2007; De Palma et al., 2010; Iebba et al., 2011; Sellitto et al., 2012). Mainly, a reduction in *Bifidobacterium* population diversity in CD patients was also found (Sanz et al., 2007; Collado et al., 2008), along with an increase of *Bacteroides* and *Clostridium leptum* groups (Collado et al., 2008).

Gluten exerts negative effects in terms of immunological response in CD; moreover, there are some evidences on possible changes induced on the colon microbiota. Bevilacqua et al. (2016) used the batch cultures approach to study the effect of the supplementation of either control or GFB on the microbiota of healthy and celiac volunteers. They found that the addition of control bread to the batches containing the microbiota of celiac volunteers determined a strong and significant decrease of BIF. On the other hand, the supplementation of GFB induced a partial restoration of BIF and lactobacilli, with a change of the whole system (microbiota+SCFA). These results were found within 48 h; thus, in the present study stool samples were collected from celiac and healthy volunteers, in order to better understand the differences in microbiota composition and metabolic activity induced by GFB after a prolonged supplementation and in a system able to simulate the whole colon.

Significant increases in bifidobacterial numbers were observed in response to GFB in the fecal microbiota of healthy and celiac donors. BIF are recognized as one of the most important bacterial groups associated with human health, providing beneficial effects in the large intestine (Gibson and Wang, 1994; Russel et al., 2011); the positive effect on bifidobacterial, as well as the slight effect on the lactic acid bacteria in the fecal microbiota of the celiac donors 1, confirmed previous results (Bevilacqua et al., 2016).

The positive effect of GFB on some bacteria could be attributed to the changes in the secondary and the tertiary structures of the proteins, as suggested by SDS page, the increase in cysteine amounts, and protein aggregation at SEM (Lamacchia et al., 2016; Landriscina et al., 2017). These changes could be responsible of the reduction of the cross-reactivity toward some antibodies (Landriscina et al., 2017), as well as on a different exposition and arrangement of charges, as postulated previously (Bevilacqua et al., 2016). This different arrangement of charges could be the primary factor leading to a positive effect on some Gram-positive bacteria, like BIF.

The GFB fermentation also increased *Clostridium* cluster XIVa. This cluster contains saccharolytic species which can produce large concentrations of beneficial SCFAs from sugars, such as butyrate. Clostridial clusters IV and XIVa have gained a lot of attention during the last years because of their contribution to gut homeostasis, by preserving gut barrier functions and exerting immunomodulatory and anti-inflammatory properties (Velasquez-Manoff, 2015). In addition, some species of the cluster, like *Faecalibacterium prausnitzii*, could produce anti-inflammatory peptides (Quévrain et al., 2016).

The SCFAs produced by gut microbiota in the colon have an important role. Butyric acid is often associated as an energy source for the epithelial cells and acetic acid plays an important role in controlling inflammation and controlling pathogen invasion (Russel et al., 2013). In addition, other effects attributed to butyrate are the improvement of the gut barrier function by stimulation of the formation of mucin, antimicrobial peptides, and tight-junction proteins, the interaction with the immune system and the reduction of the oxidative stress in the colon (Rivière et al., 2016). Therefore, the increase of butyrate found in the vessel 2 inoculated with the microbiota of a celiac donor could be of interest and deserves further efforts and experiments.

Moreover, the increase of acetic and propionic acids is interesting as they also act as anti-inflammatory compounds, could play a role in the stimulation of the immune system, and decrease the pH of the colon (indirect antimicrobial effect) (Rivière et al., 2016).

The multivariate approaches and the NIPALS algorithm also suggest that the effect of GFB could also rely on the initial equilibrium of the fecal microbiota and the positive effect could result in a different modulation of the microorganisms and SCFA; in the present study, in the fecal microbiota of a celiac donor a pronounced effect on BIF was found, while the fecal microbiota of the other celiac donor experienced a significant effect on *Lactobacillus*/*Enterococcus*.

In conclusion, this *in vitro* work provides encouraging findings supporting the utilization of GF products with a positive effect on the fecal microbiota and SCFAs from celiac donors. Generally, GFB induced increases in bifidobacterial counts and a positive modulation of SCFAs, with an increase of butyrate, acetate, and propionate. In addition, the use of multivariate approaches showed that the supplementation of GFB could result in a different modulation of the fecal microbiota and SCFA, as a function of initial equilibrium of the microbiota.

The experiments were done on fecal slurries from two healthy and two celiac donors, thus the results are as a kind of preliminary evidence and should be confirmed and validated on a larger number of samples and by an *in vivo* human intervention study. However, the most important achievement of this paper is a new horizon to be explored and studied: the possibility of the modulation of colon microbiota by GFB.

## Author Contributions

AB, MC, MS, CL, and AC conceived this study; AC and CL provided valuable input for this study’s design and data analyses; TB-M, IG-S, and LL performed the fermentation analyses; MC, LP, and MS helped in the analysis; AB performed the statistical analyses; TB-M, AB, and AC wrote the paper; and all authors edited and approved the final manuscript.

## Disclaimer

This publication reflects only the author’s view and the Agency is notresponsible for any use that may be made of the information it contains.

## Conflict of Interest Statement

The authors declare that the research was conducted in the absence of any commercial or financial relationships that could be construed as a potential conflict of interest. The reviewer LV and handling Editor declared their shared affiliation, and the handling Editor states that the process nevertheless met the standards of a fair and objective review.

## Footnotes

1 http://www.casillogroup.com/en/home-eng.html
